# Acute abdomen due to spontaneous splenic rupture as the first presentation of lung malignancy: a case report

**DOI:** 10.1186/1752-1947-5-444

**Published:** 2011-09-07

**Authors:** Angelos Kyriacou, Nolan Arulraj, Haren Varia

**Affiliations:** 1 Department of Medicine, Whinney Heys Road, Blackpool Victoria Hospital, Blackpool, FY3 8NR, UK; 2 Department of Radiology, Whinney Heys Road, Blackpool Victoria Hospital, Blackpool, FY3 8NR, UK

## Abstract

**Introduction:**

Spontaneous splenic rupture is well recognized in the context of hematological malignancies (lymphoproliferative and myeloproliferative disorders); a few case reports have also linked solid tumors, such as pancreatic and liver cancer, with the occurrence of spontaneous splenic rupture. This is the first case report of lung cancer as a likely cause of spontaneous splenic rupture.

**Case presentation:**

A 61-year-old Caucasian woman presented to our hospital with non-specific symptoms. She developed an 'acute' abdomen and went into a state of shock within twelve hours of her presentation. She was diagnosed with spontaneous splenic rupture with radiology and following a laparotomy. She made an uneventful recovery postoperatively and was simultaneously found to have a bronchial adenocarcinoma.

**Conclusion:**

Spontaneous splenic rupture is a potentially fatal but often unrecognized cause of acute abdomen. It should be routinely considered in the differential diagnosis of acute ('surgical') abdomen and when present it should be promptly dealt with, most commonly with a laparotomy. Once the diagnosis is confirmed there should be an aggressive drive to identify an underlying etiology; malignancy is the commonest culprit. Solid tumors should be considered as underlying causes despite being less common than hematological neoplasms. This case report demonstrates lung malignancy as an underlying precipitating cause of spontaneous splenic rupture.

## Introduction

Splenic rupture is a rare albeit potentially catastrophic event that causes an 'acute' abdomen and hemodynamic instability. It should be urgently investigated, diagnosed and treated, often with splenectomy and less often with conservative management or with splenic artery embolization.

Splenic rupture can be divided into traumatic (or non-spontaneous) and atraumatic (or spontaneous). Diagnostic criteria were developed by Orloff and Peskin in 1958 [1] for spontaneous rupture which requires that all of the following conditions are met: (a) no history of trauma or unusual effort that could rupture the spleen; (b) no evidence of disease in the organs, other than the spleen, which is known to affect the spleen adversely causing pathological rupture; (c) no evidence of perisplenic scarring or adhesions suggestive of previous rupture or trauma; (d) other than hemorrhage the spleen should be normal on both gross inspection and histology; (e) clotting studies should be normal; (f) other criteria including no significant rise in viral antibody titers in acute or convalescent sera.

**Figure 1:**
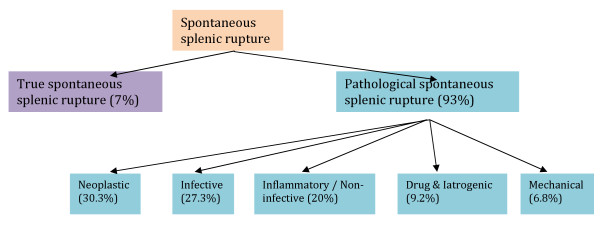
**The causes of pathological rupture, adapted from Renzulli *et al***. [2].

Spontaneous splenic rupture (SSR) can be subdivided further into true and pathological rupture corresponding to normal and pathological appearances of the spleen on histological examination. Therefore, in essence, the original Orloff and Peskin criteria describe 'true' SSR. A systematic literature review [2] of 845 cases of atraumatic splenic rupture between 1980 and 2008 found that the former was much rarer than the latter (7% true versus 93% pathological splenic rupture). The causes of pathological rupture are shown in Figure 1.

Hematological malignancies (mainly non-Hodgkin's lymphoma, chronic myeloid leukemia and acute lymphoblastic leukemia) comprise the majority of the neoplastic causes, with solid tumors rarely reported in the literature (such as hepatocellular [3] and pancreatic carcinoma [4]).

The investigation of choice for confirming the diagnosis is computed tomography (CT) of the abdomen [5], which has a sensitivity and specificity of 90-95%. Splenectomy remains the treatment of choice for splenic rupture with hemodynamic instability, and should be urgently performed once the diagnosis is confirmed. Proximal splenic artery embolization can be performed to treat SSR [6] in a hemodynamically stable patient but requires an interventional radiology department with expertise in the relevant procedure and close monitoring of vital signs and hemoglobin concentration, ideally in an intensive care setting. The potential benefits of this technique include a reduced risk of repeated hemorrhage with preservation of the splenic tissue and reduced levels of postoperative sepsis [6,7].

In a systematic review by Renzulli *et al. *[2] splenectomy was performed in 84.1% of cases; the rest were either treated conservatively (14.7%) or with organ-preserving surgery (1.2%). Whenever possible, the underlying cause should be treated. Factors that increase mortality include underlying neoplastic disease, splenomegaly and increasing age [2].

### Case Presentation

A 61-year-old Caucasian woman, previously in good health, presented with a three-day history of feeling generally unwell with dizziness, vomiting, abdominal, left lower chest pain and shoulder pain. She denied any sore throat, feeling feverish or other symptoms suggestive of an influenza-like illness. There was no cough or sputum production. There was nothing in the history to suggest a recent viral or other infective process including human immunodeficiency virus or acquired immune deficiency state and no history of any trauma or injury. She had no significant previous medical or surgical history and was not taking any medications. There was no background or family history of cancer, hematologic or clotting disorders. She was a smoker of approximately 40 pack years and had unlimited exercise tolerance. She worked as a nurse and there was no history of previous exposure to asbestos or other occupational hazards.

On examination she was ill-looking, conscious and orientated with a blood pressure 89/49 mmHg, heart rate of 72 beats per minute, saturations 97% on a non-rebreather mask and temperature of 36.9°C. The admission examination revealed normal cardiac examination and bibasal inspiratory crepitations with left upper quadrant and epigastric tenderness on abdominal examination.

**Figure 2:**
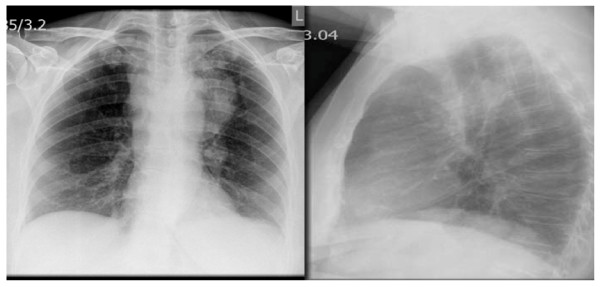
**Large left anterior mediastinal mass with prominent left hilum**.

Her blood work-up on admission showed a hemoglobin level of 11.4 g/L, white cell count of 19 × 10^9^/L with neutrophils of 17 × 10^9^/L and a C-reactive protein of 7 mg/L. Urea and electrolytes, liver function and coagulation tests were in the normal range. A blood film examination did not reveal any atypical lymphocytes or other abnormalities. A posteroanterior and lateral chest radiograph was performed (Figure 2).

Hemodynamic stability was achieved following rehydration with intravenous fluids. However, eight hours after admission she developed an acute abdomen with clinical signs of shock. Her blood pressure was 88/52 mmHg and heart rate 105 beats per minute. Further to that her oxygen saturations were 91% on a non-rebreather mask with a Glasgow coma scale of 14/15 (E = 4, V = 4, M = 6). Inspection revealed skin and conjunctival pallor. Clinical examination revealed new abdominal signs of generalized abdominal tenderness with guarding, rigidity and absent bowel sounds, consistent with an acute abdomen. A repeat test showed her hemoglobin level had dropped to 5.0 g/L.

**Figure 3:**
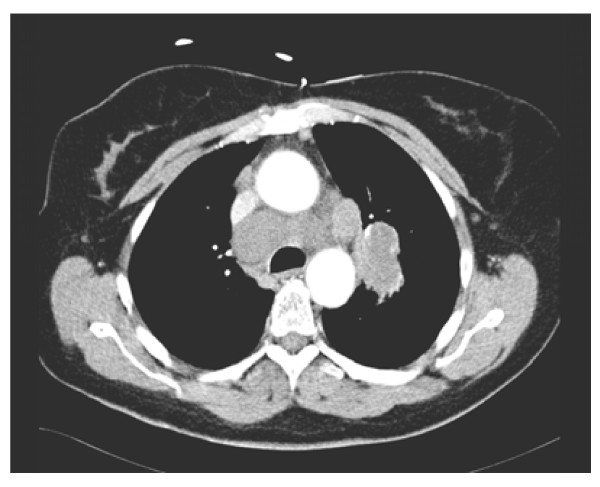
**Left hilar mass measuring 5 cm with associated significant mediastinal adenopathy and a 3 cm upper right paratracheal node**.

**Figure 4:**
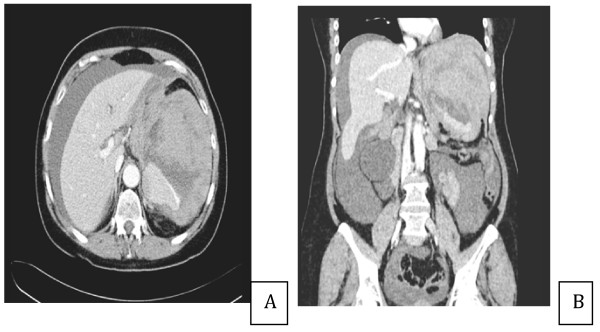
**A and B. Large splenic hematoma**. Inferomedial areas of high attenuation suggestive of active bleeding. Compressed and slit-like inferior vena cava indicative of hemodynamic instability. Extensive ascites also present.

She was stabilized with multiple blood transfusions and underwent an urgent computer tomography scan of her thorax and abdomen, which showed an 8.5 × 3.6 cm left hilar mass with extensive mediastinal adenopathy, bibasal small effusions and consolidation, and a large splenic hematoma of 15 × 12 cm with high attenuation suggestive of active bleeding (Figure 2, Figure 3 and 4). There was a lytic area affecting her T9 vertebra, which likely represented metastases rather than wedge fracture, but there were no abnormalities or neoplastic disease affecting the intra-abdominal organs.

Pneumococcal and meningococcal vaccines were administered and our patient was then promptly taken to theater for laparotomy. On examination of her internal organs at laparotomy, other than hemorrhage and rupture of her spleen, there was no other gross abnormality and no evidence of disease in her other intra-abdominal organs. She spent two days being ventilated in our intensive care unit and subsequently made a full recovery from the surgery. She was commenced on life-long penicillin V and was subsequently discharged home a week after admission, fully mobile and independent in terms of her activities of daily living.

Her splenic histology was negative for hematologic or other malignancy and no other pathology was identified. She then underwent a bronchoscopy; transbronchial needle aspiration revealed numerous malignant nodes consistent with non-small cell carcinoma. Histology of bronchial biopsies confirmed invasive adenocarcinoma. This was consistent with likely stage IV in view of the likely bone metastases found on CT. She was referred to the local oncologist and her case was discussed at the lung cancer multidisciplinary team meeting. In view of the diagnosis and staging, a palliative treatment pathway was agreed from the outset and our patient received palliative chemotherapy. She died five months after her presentation.

## Discussion

To the best of our knowledge, this is the first case report of SSR in association with lung cancer. Lung cancer with splenic rupture has been reported either in the context of splenic metastasis [8], after the initiation of chemotherapy in a patient with a splenic hamartoma (splenoma) [9] or after pegfilgrastim was given to prevent neutropenia [10]. Few case reports have previously described the occurrence of splenic rupture in patients with known lung cancer; usually in widely disseminated metastatic disease. The incidence of isolated splenic metastasis ranges from 0-26% of all patients with splenic metastasis from a literature review [11]. The length of time until diagnosis of splenic metastasis from the diagnosis of a primary lung cancer ranges between 0-8 years [11]. However, our case is of interest because there were no metastatic deposits and the lung cancer presented itself with SSR, as opposed to respiratory symptoms.

The mortality rate for conservatively managed spontaneous splenic rupture was 22% at 30 days; mortality was 30% versus 5% for malignant and benign underlying disease, respectively [12]. The traumatic splenic rupture postoperative mortality rate was 18% [12].

It is possible that our patient had two concurrent, unrelated pathologies: bronchial adenocarcinoma and a true SSR. However, given the well-described association between neoplastic disease and SSR, the above case reports linking the two conditions and the rarity of true spontaneous splenic rupture, it is likely that the two conditions are linked with the former precipitating the latter.

## Conclusion

Our case illustrates the need for extra vigilance to make the diagnosis of splenic rupture in the context of a lack of trauma. The exact pathophysiology that links lung cancer with SSR (in the absence of splenic metastasis or use of chemotherapy or pegfilgrastim) remains unclear. The possible explanations include a hypercoagulable state secondary to the underlying malignancy.

In summary, this case demonstrates that lung cancer could potentially precipitate SSR; it could even present itself as SSR as in our patient. This association is of relevance to a wide variety of health care professionals including acute and general physicians, general surgeons, oncologists, radiologists and others. The other important learning point from this case is that although SSR is a rare cause of acute abdomen, we should bear in mind that it is fatal if misdiagnosed and left untreated; it should therefore be considered in the differential diagnosis of a wide range of medical and surgical conditions.

## Consent

Written informed consent was obtained from the patient for publication of this manuscript and any accompanying images. A copy of the written consent is available for review by the Editor-in-Chief of this journal.

## Competing interests

The authors declare that they have no competing interests.

## Authors' contributions

AK contributed to the medical care of this patient, the manuscript preparation and revision, and also performed the literature review. NA contributed to the medical care of this patient, the manuscript preparation and revision. HV contributed to the radiological review of the case report and the manuscript revision. All authors read and approved the final manuscript.
